# Supplementary figures and images for: In vitro and in vivo characterization of a recombinant rhesus cytomegalovirus containing a complete genome
Source: PLoS Pathog. 2020 Nov 24;16(11):e1008666. doi: 10.1371/journal.ppat.1008666 (PMC7723282; doi:10.1371/journal.ppat.1008666)

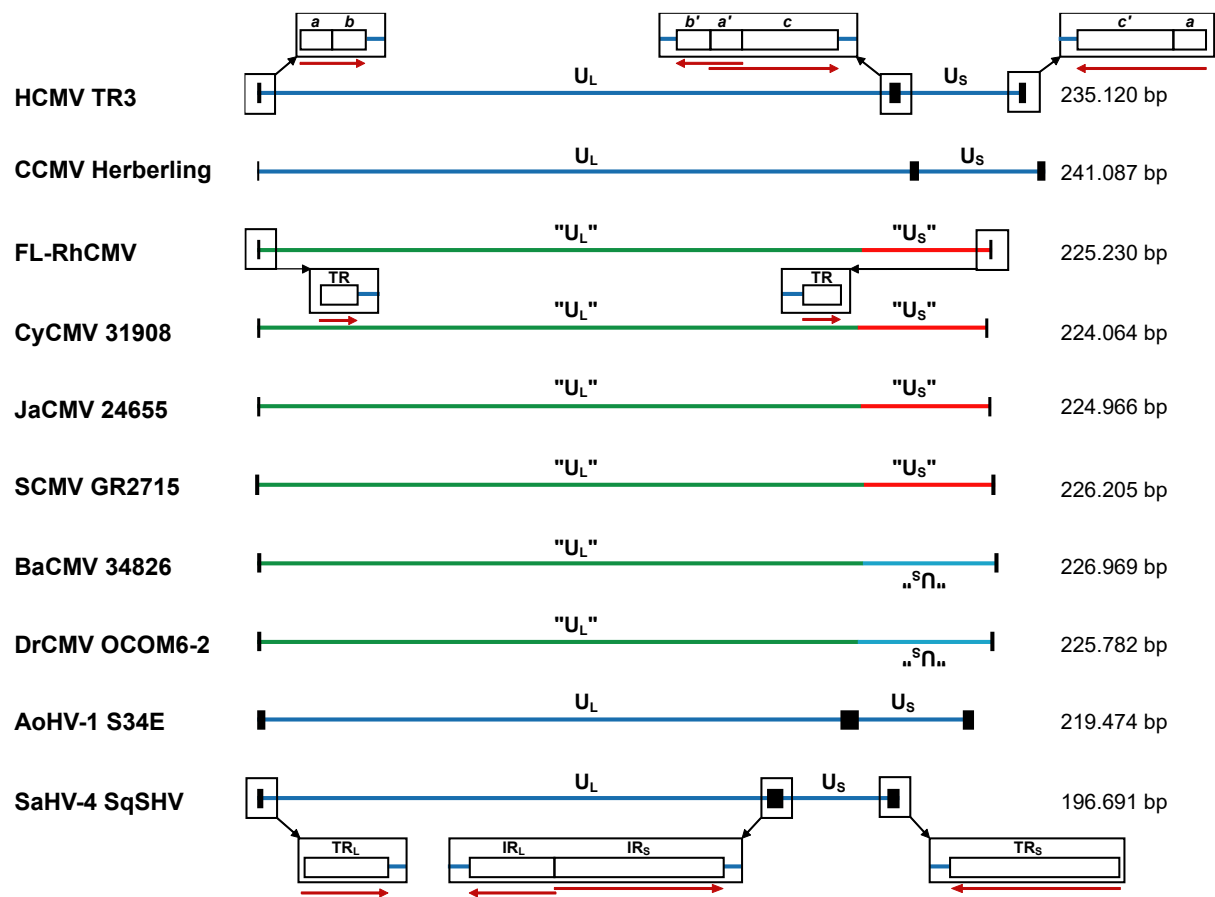

Supplement: S1 Fig — The genome of HCMVs and the closely related CCMV comprise two unique coding regions (UL and US) that are separated by an internal repeat region and flanked by terminal repeats. The repeat regions consist of the three repeated sequence units a, b and c that form overlapping inverted repeats in the form ab-UL-b’a’c-US-c’a. The HCMV genome can re-arrange to four different isomers varying in the relative orientation of the UL and US regions to one another [109]. Intriguingly, while the UL and US regions can still be identified in old world NHP CMVs, the repeat organization is vastly different. The terminal direct repeats in these species are short while the internal repeats are completely missing resulting in a single isomer that has been fixed during evolution. All Asian NHP CMVs and the African green monkey (Simian) CMV (SCMV) occur in the same isomeric form whereas the US region appears in the opposite orientation to the UL region in the closely related BaCMV and DrCMV. New world (NW) CMVs retained a genome organization with terminal and internal repeats similar HCMV, but the repeats are organized as non-overlapping inverted repeats flanking the UL and US regions, allowing for isomerization. (PDF) [file ppat.1008666.s001.pdf]

**A**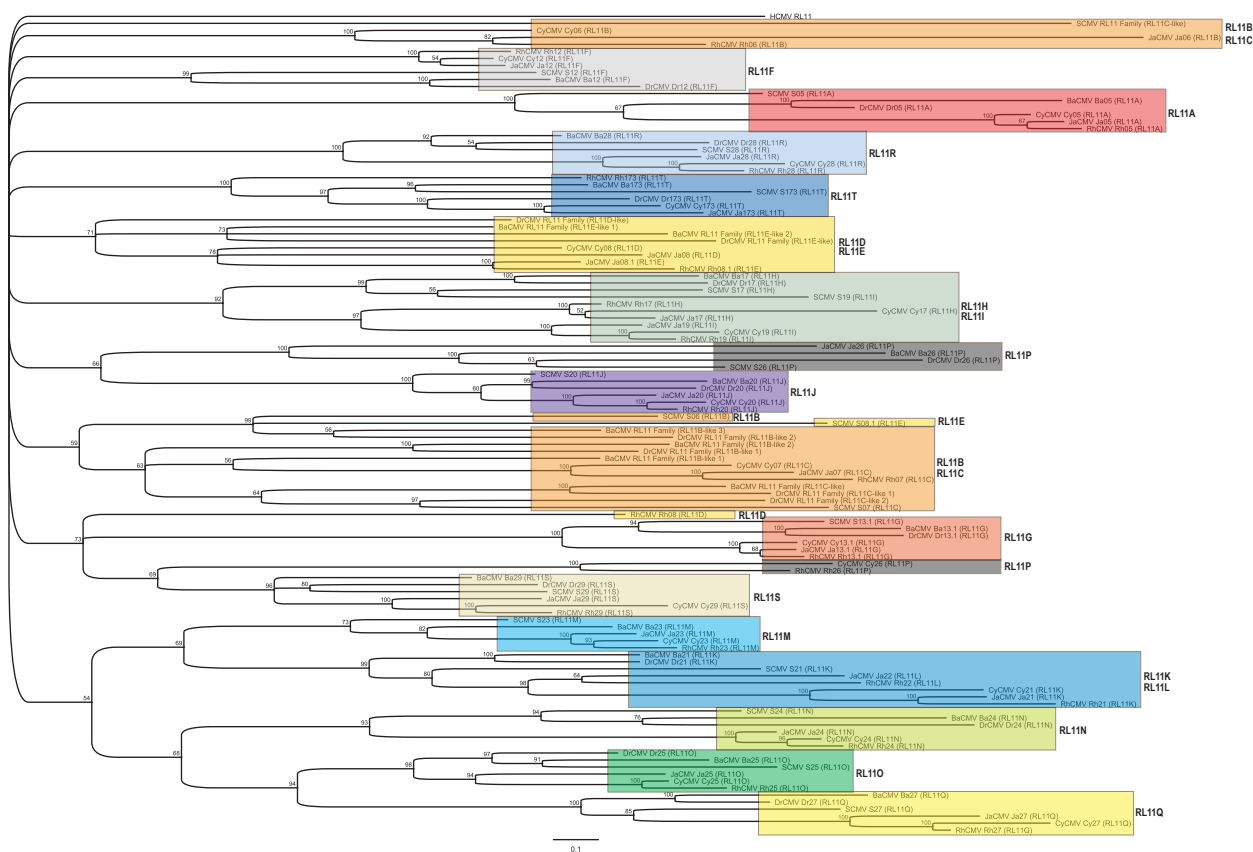**B**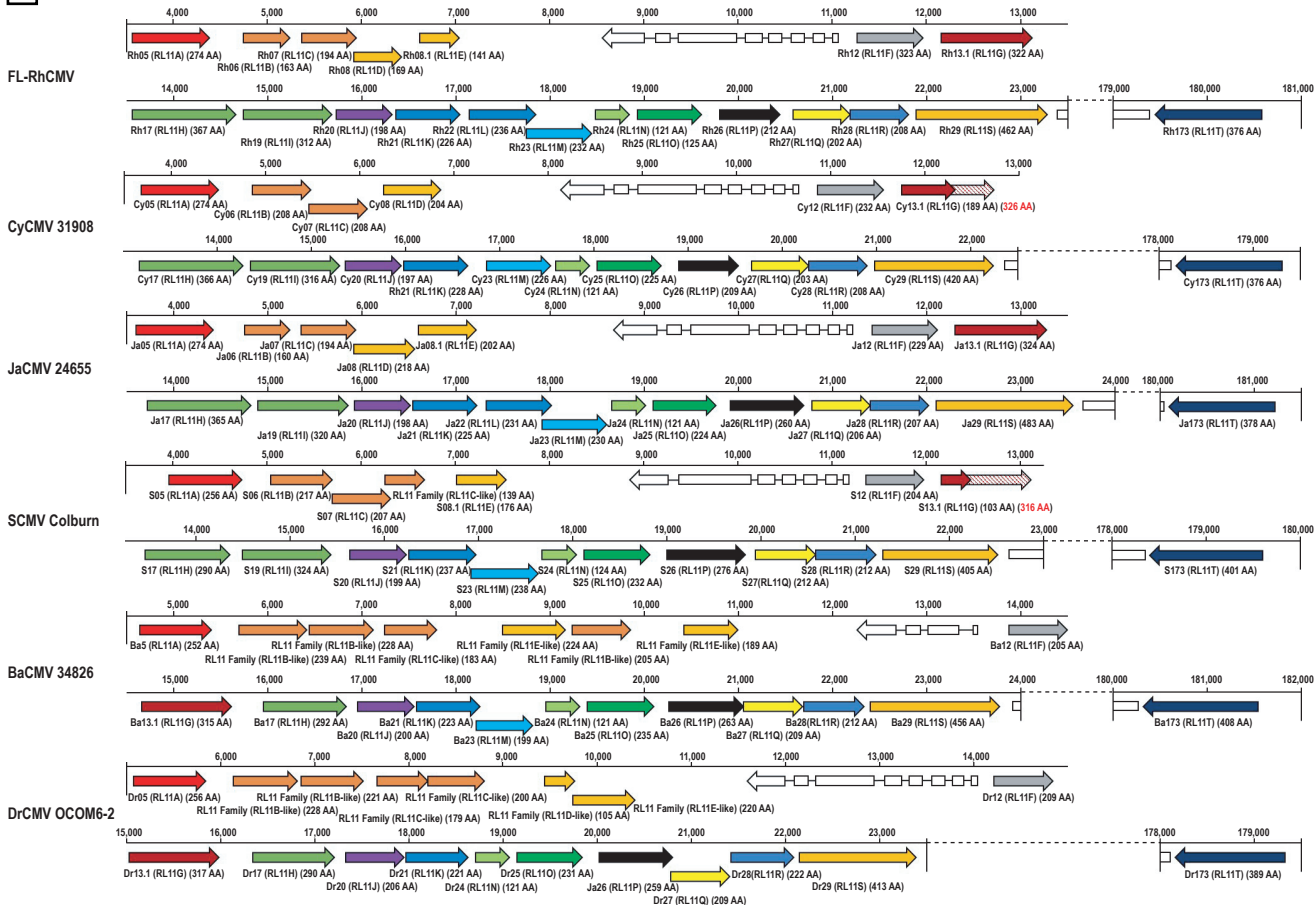

Supplement: S2 Fig — A) Phylogenetic tree of RL11 family genes from representatives of each NHP CMV species. B) ORF structure of the RL11 family genes in each NHP CMV species. Each gene is color-coded using the same colors as in A) showing the presence/absence of each ORF in a given NHP CMV species. (PDF) [file ppat.1008666.s002.pdf]

**A**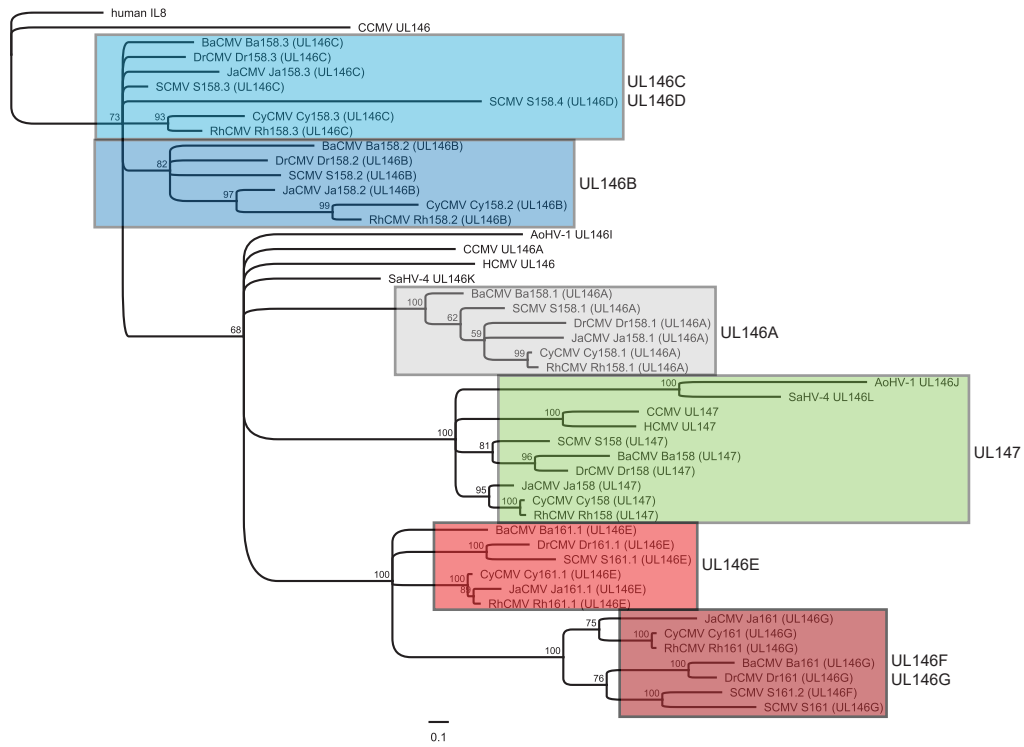**B**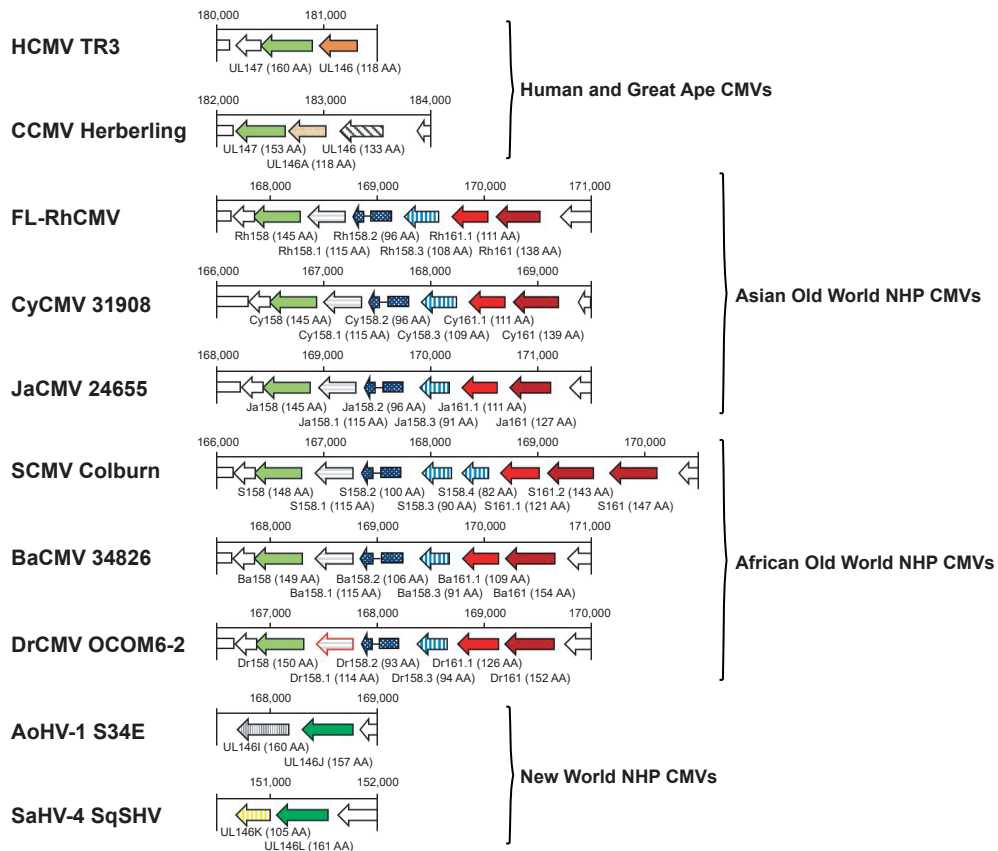

Supplement: S4 Fig — A) Phylogenetic tree based on the protein sequences of NHP CMV genes homologous to HCMV chemokine-like genes UL146 and UL147 from representatives of each NHP CMV species. B) ORF structure of the UL146/147 family genes in each NHP CMV species. Each gene is color-coded using the same colors as in A) showing the presence/absence of each ORF in a given NHP CMV species. (PDF) [file ppat.1008666.s004.pdf]

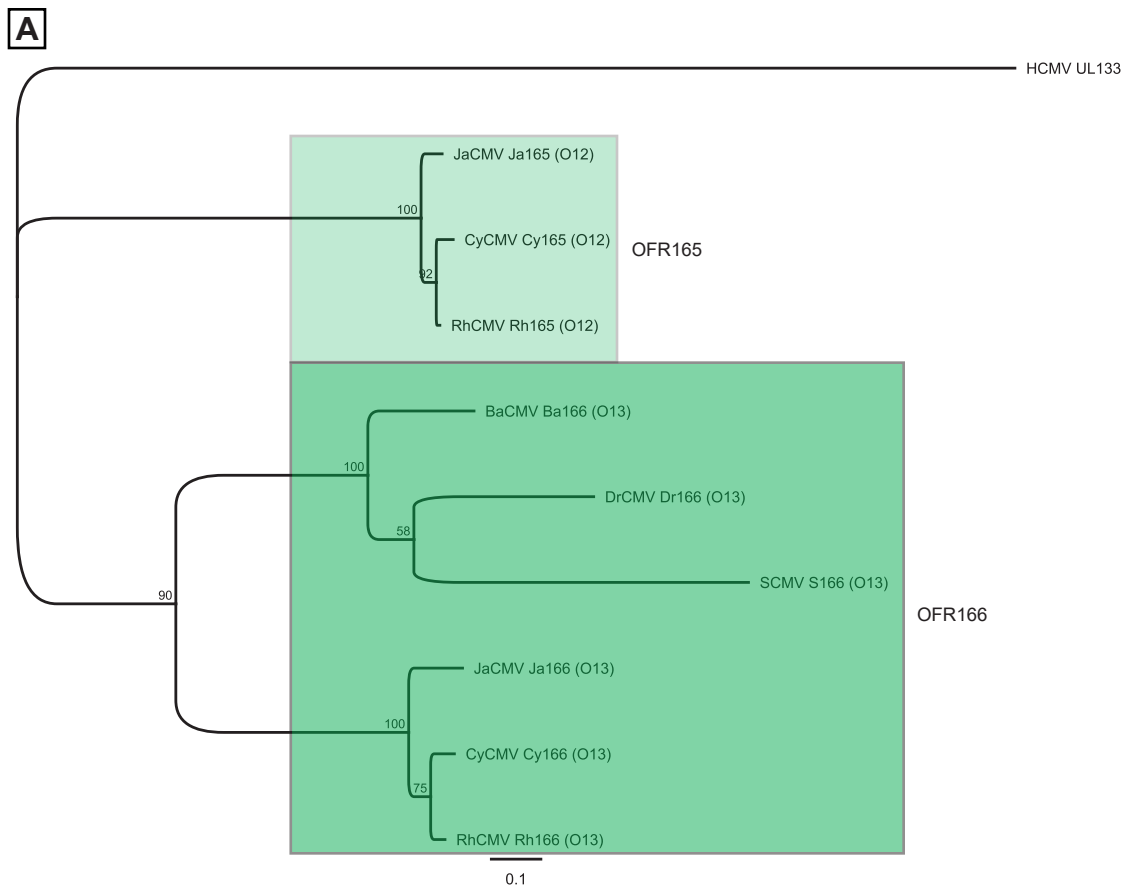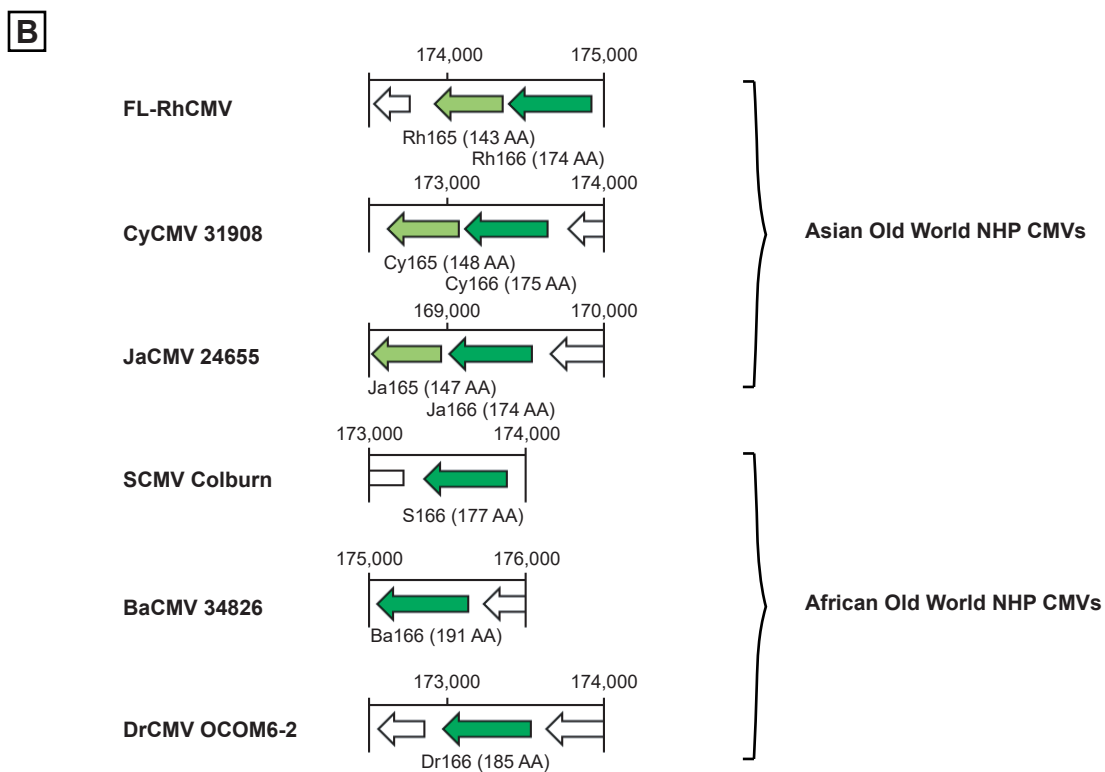

Supplement: S5 Fig — A) Phylogenetic tree based on the protein sequences of Rh166 family genes from representatives of each NHP CMV species. B) ORF structure of the Rh166 family genes in each NHP CMV species. Each gene is color-coded using the same colors as in A) showing the presence/absence of each ORF in a given NHP CMV species. (PDF) [file ppat.1008666.s005.pdf]

**A**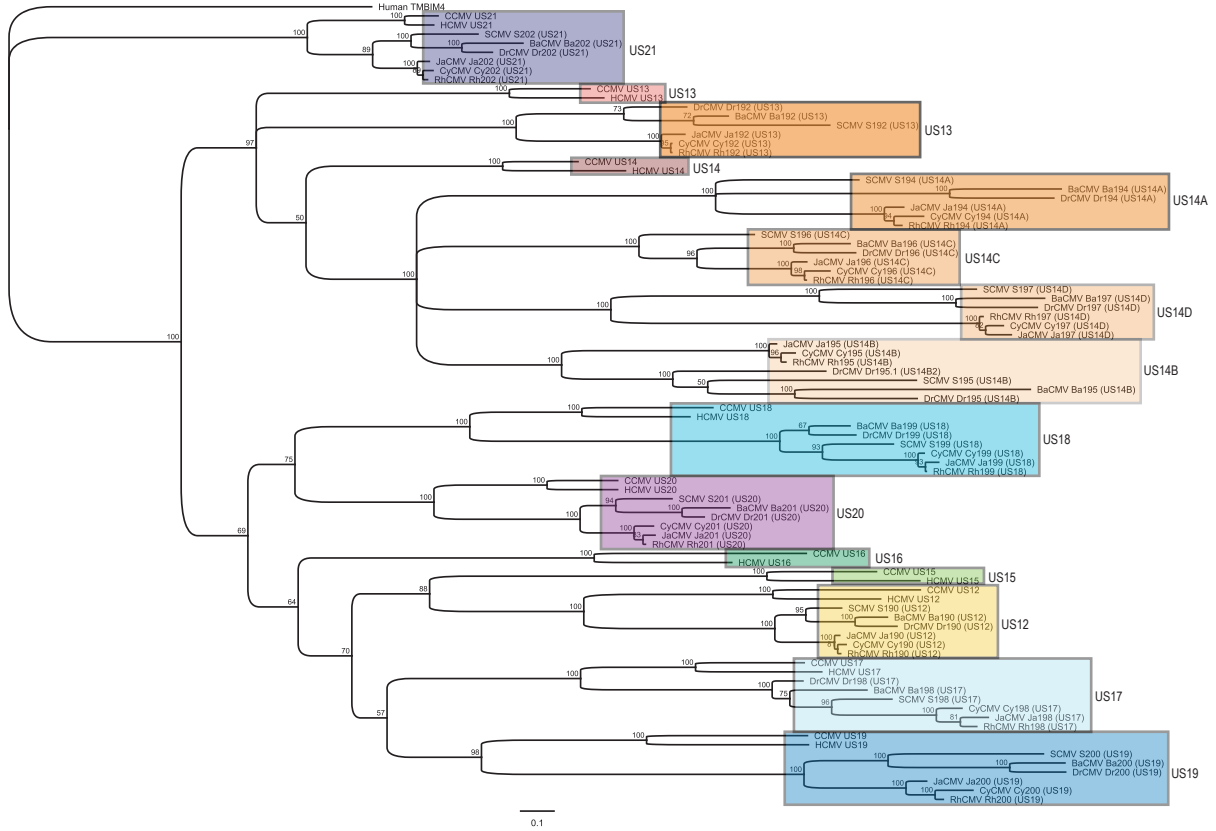**B**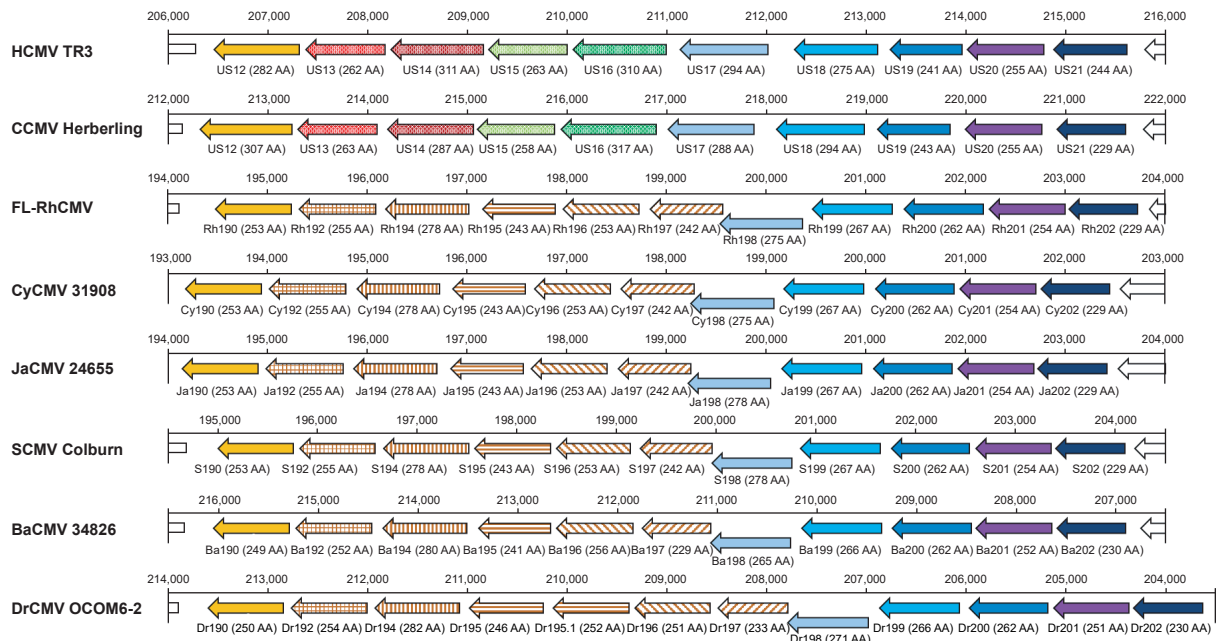

Supplement: S6 Fig — A) Phylogenetic tree based on the protein sequences of NHP CMV genes homologous to the HCMV US12 family encoding seven transmembrane proteins from representatives of each NHP CMV species. B) ORF structure of the US12 family genes in each NHP CMV species. Each gene is color-coded using the same colors as in A) showing the presence/absence of each ORF in a given NHP CMV species. (PDF) [file ppat.1008666.s006.pdf]

**A**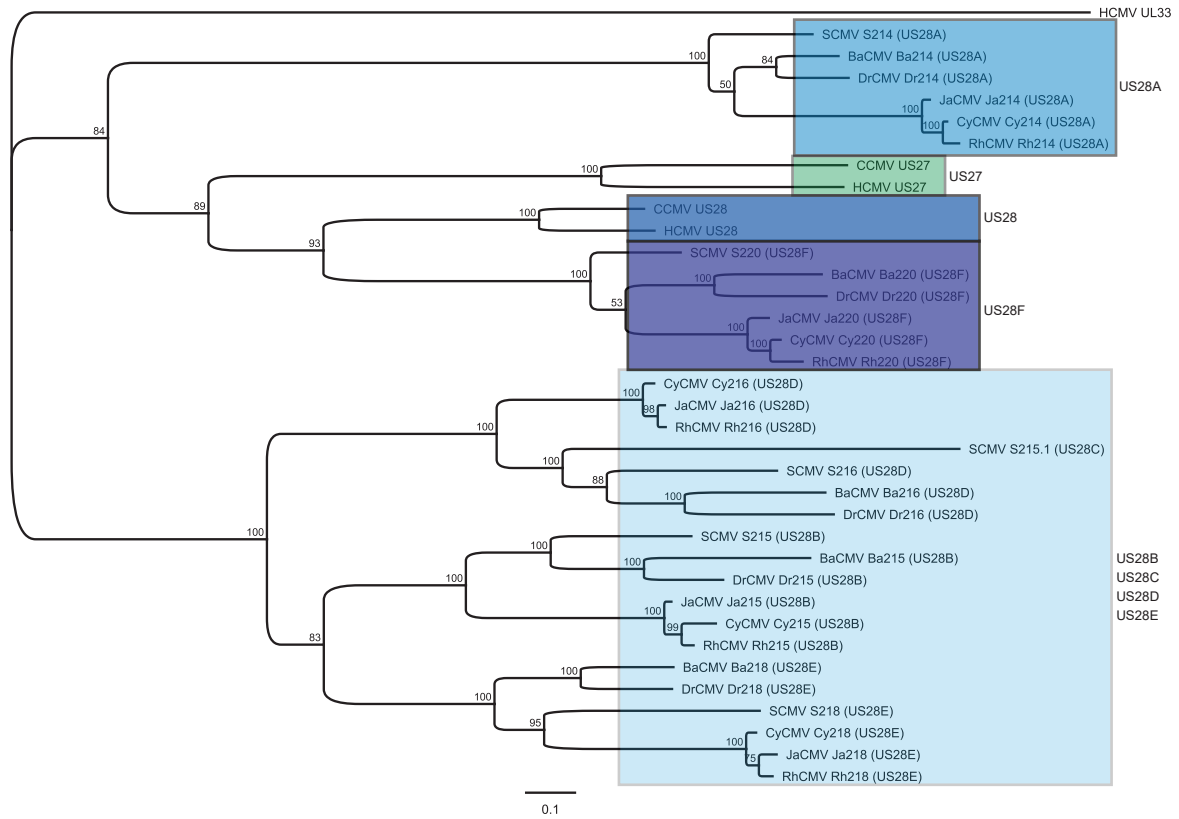**B**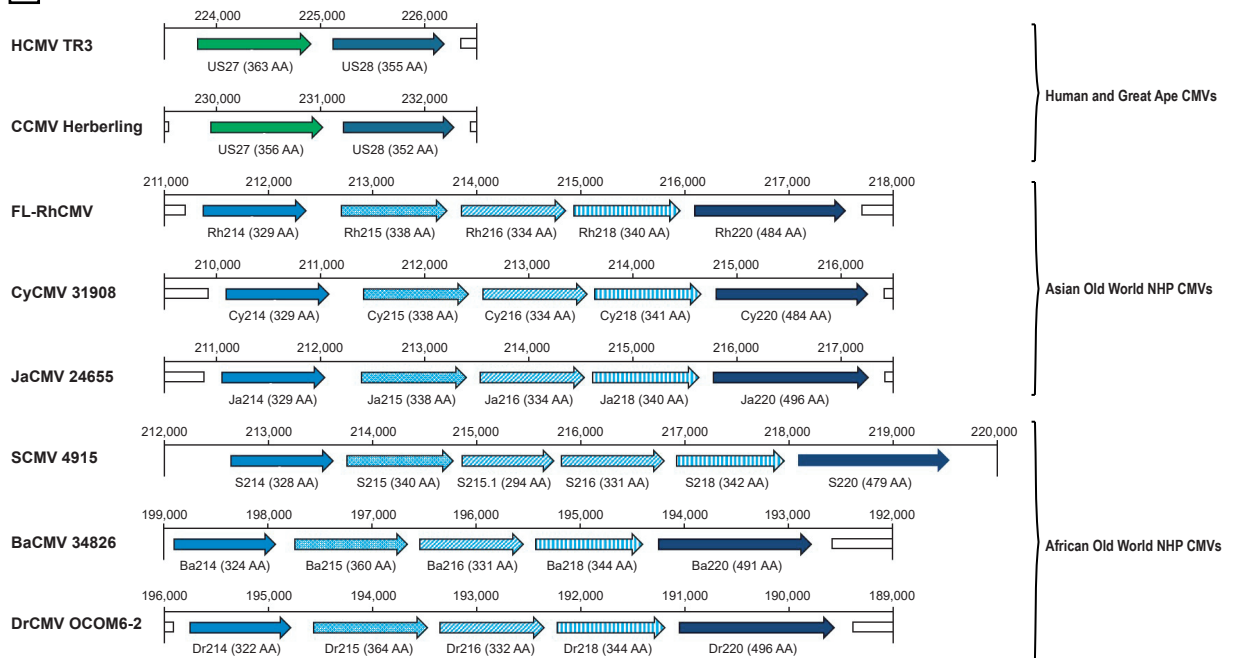

Supplement: S7 Fig — A) Phylogenetic tree based on the protein sequences of NHP CMV genes homologous to HCMV US28 encoding G-protein coupled receptors from representatives of each NHP CMV species. B) ORF structure of the US28 family genes in each NHP CMV species. Each gene is color-coded using the same colors as in A) showing the presence/absence of each ORF in a given NHP CMV species. (PDF) [file ppat.1008666.s007.pdf]

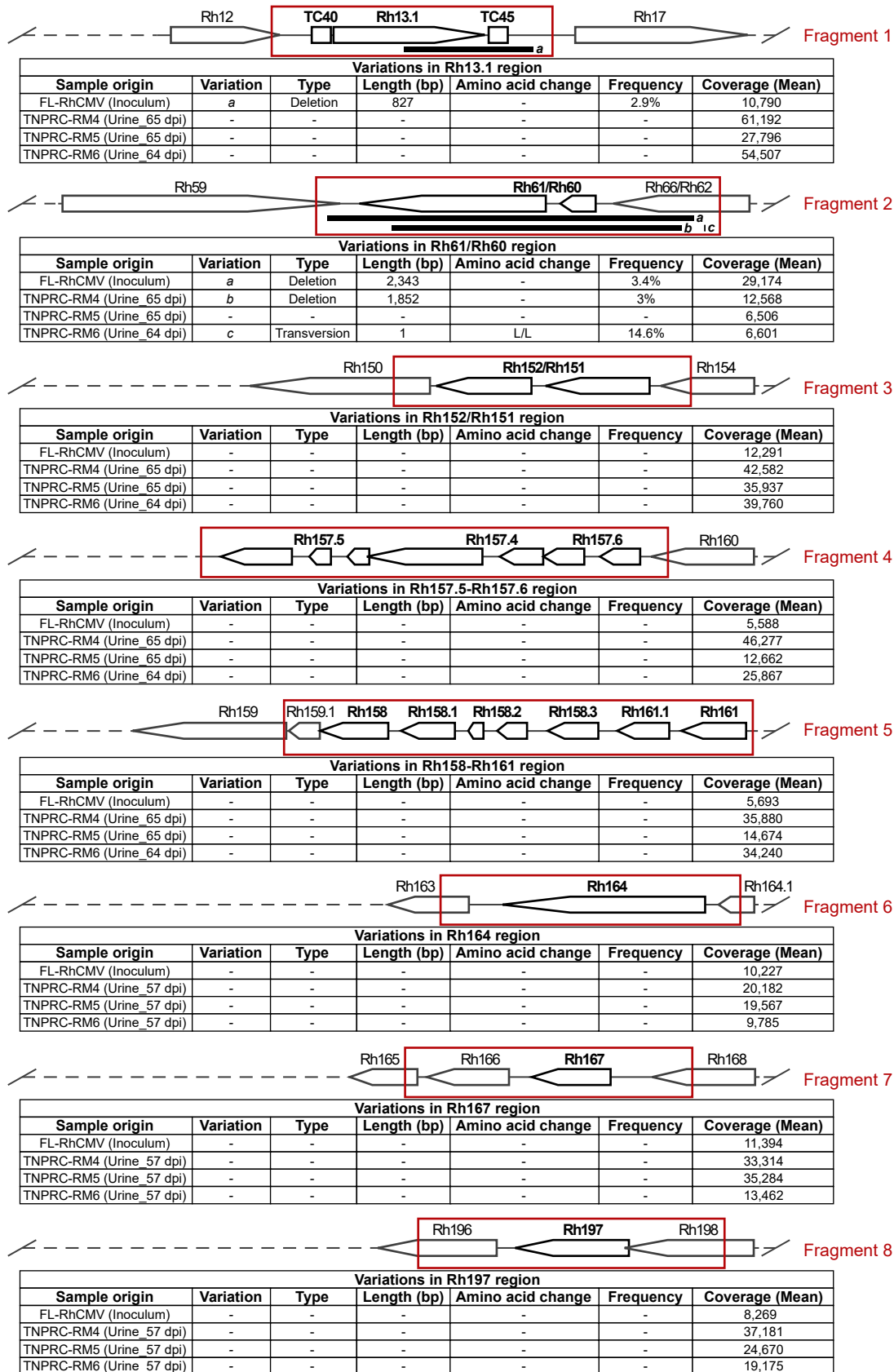

Supplement: S8 Fig — Schematic overview depicting the eight DNA fragments (red boxes) that were amplified by PCR from urine samples collected at the indicated timepoints from RhCMV naïve RM infected with FL-RhCMV/Rh13.1apt. PCR amplifications of genome fragments from the viral inoculum were included as a pre-infection controls. All viral ORFs encoded in the targeted genomic region are shown in black boxes. The position and frequency of each putative variation was determined by NGS within each corresponding enriched gene region. The location of each variation with a frequency of 1% or greater relative to the mean coverage of that particular gene region is graphed under the map of the corresponding locus. All variation are labeled with lower case letters ranked by their position in the genomic fragment. All results are shown in comparison to the clonal FL-RhCMV/Rh13.1apt BAC sequence. (PDF) [file ppat.1008666.s008.pdf]

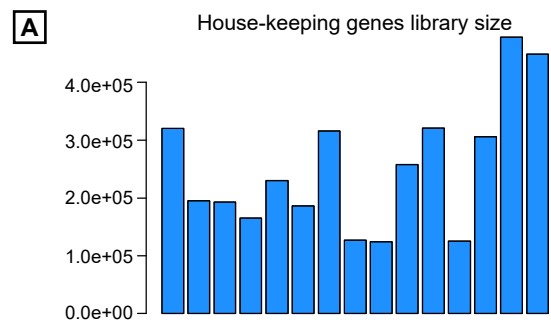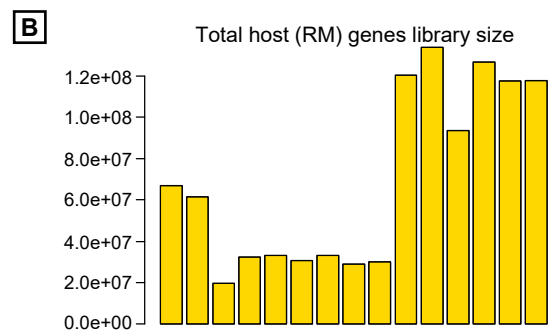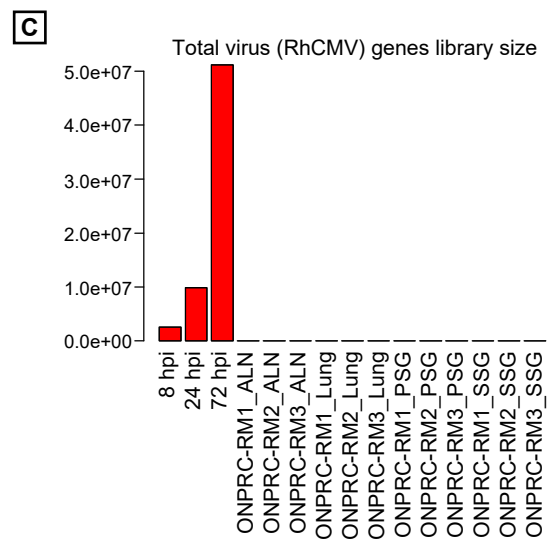

Supplement: S9 Fig — To account for equivalency across the RNA-seq samples, A) background expression was assessed using house-keeping genes ("ACTG1", "RPS18", "MRPL18", "TOMM5", "YTHDF1", "TPT1", "RPS27") [110] which did not identify a specific trend across samples. Next, B) host (RM) library size was assessed across samples which showed higher overall transcript levels that were tissue specific to the salivary glands. However, this difference did not correlate with the viral gene expression levels. Finally, C) we found that all three in vitro samples had a higher number of total viral reads than tissue biopsies. (PDF) [file ppat.1008666.s009.pdf]

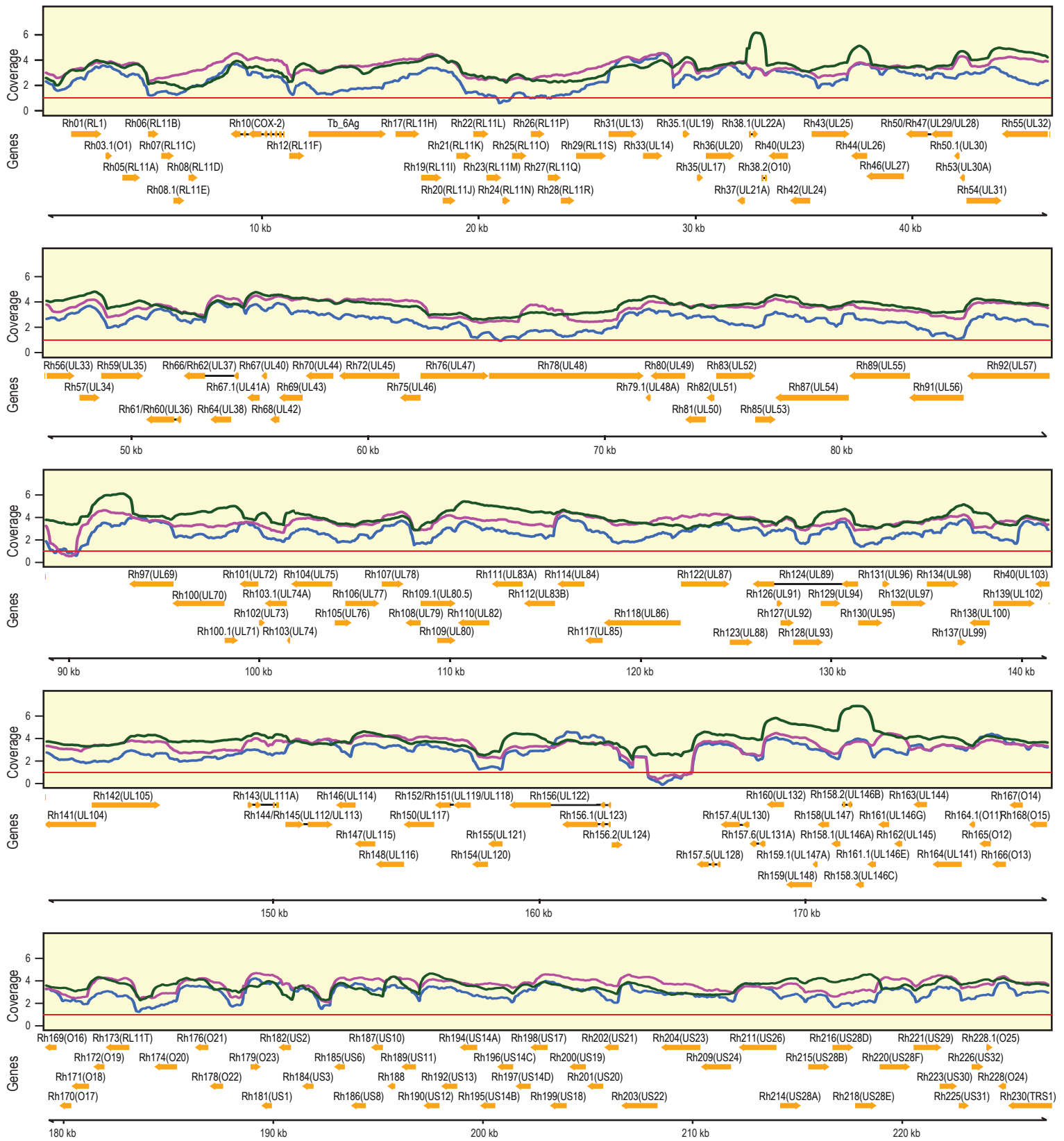

Supplement: S10 Fig — Sequence coverage of FL-RhCMVΔRh13.1/TB6Ag in cultured fibroblasts, infected in vitro and sampled at three timepoints post-infection (8, 24, 72 hours). Coverage per base is plotted with a log10 scale. Colors: blue = 8hpi, magenta = 24hpi, green = 72hpi. (PDF) [file ppat.1008666.s010.pdf]
