# Supplementary material for: In vitro and in vivo characterization of a recombinant rhesus cytomegalovirus containing a complete genome
Source: PLoS Pathog. 2020 Nov 24;16(11):e1008666. doi: 10.1371/journal.ppat.1008666 (PMC7723282; doi:10.1371/journal.ppat.1008666)
Supplement: S1 Table — (DOCX) [file ppat.1008666.s011.docx]

| **ORF** | **Alteration from original to current annotation** | **ORF in RhCMV 68-1 (original annotation)** | **ORF in RhCMV 68-1 (current annotation)** |
| --- | --- | --- | --- |
| TR | Newly annotated repeat. | / | 764 bp |
| Rh01 (RL1) | Different start codon annotated. | 1527 bp | 1404 bp |
| Rh03.1 (O1) | Newly annotated ORF. | / | 303 bp |
| Rh04 | Deleted annotation. | 699 bp | / |
| Rh10 (COX-2) | Different start codon and additional exon annotated. | 1761 bp | 1866 bp |
| Rh24 (RL11N) | Different start codon annotated. | 390 bp | 363 bp |
| Rh26 (RL11P) | Different start codon annotated. | 849 bp | 636 bp |
| Rh36 (UL20) | Different start codon annotated. | 1347 bp | 1332 bp |
| Rh38.1 (UL22A) | Newly annotated ORF. | / | 318 bp |
| Rh38.2 (O10) | Newly annotated ORF. | / | 162 bp |
| Rh40 (UL23) | Different start codon annotated. | 939 bp | 900 bp |
| Rh50.1 (UL30) | Different start codon annotated. | 255 bp | 252 bp |
| Rh53 (UL30A) | Newly annotated ORF. | / | 240 bp |
| Rh56 (UL33) | Different start codon annotated. | 1326 bp | 1206 bp |
| Rh66/Rh62 (UL37) | Different start codon and different splice sites annotated. | 1164 bp | 1125 bp |
| Rh92 (UL57) | Different start codon annotated. | 3492 bp | 3483 bp |
| Rh101 (UL72) | Different start codon annotated. | 1032 bp | 996 bp |
| Rh103.1 (UL74A) | Newly annotated ORF. | / | 171 bp |
| Rh114 (UL84) | Different start codon annotated. | 1539 bp | 1422 bp |
| Rh144/Rh145 (UL112/UL113) | Different splice sites annotated. | 1719 bp | 1644 bp |
| Rh156.2 (UL124) | Different start codon annotated. | 444 bp | 420 bp |
| Rh158.1 (UL146A) | Different start codon annotated. | 366 bp | 345 bp |
| Rh158 (UL147) | Different start codon annotated. | 462 bp | 435 bp |
| Rh159.1 (UL147A) | Newly annotated ORF. | / | 198 bp |
| Rh160 (UL132) | Different start codon annotated. | 666 bp | 657 bp |
| Rh161 (UL146G) | Different start codon annotated. | 441 bp | 339 bp |
| Rh164 (UL141) | Different start codon annotated. | 1293 bp | 1104 bp |
| Rh179 (O23) | Different start codon annotated. | 516 bp | 489 bp |
| Rh187 (US10) | Different start codon annotated. | 681 bp | 570 bp |
| Rh189 (US11) | Different start codon annotated. | 846 bp | 693 bp |
| Rh190 (US12) | Different start codon annotated. | 783 bp | 759 bp |
| Rh195 (US14B) | Different start codon annotated. | 744 bp | 729 bp |
| Rh197 (US14D) | Different start codon annotated. | 702 bp | 690 bp |
| TR | Newly annotated repeat. | / | 764 bp |
