# Supplementary material for: In vitro and in vivo characterization of a recombinant rhesus cytomegalovirus containing a complete genome
Source: PLoS Pathog. 2020 Nov 24;16(11):e1008666. doi: 10.1371/journal.ppat.1008666 (PMC7723282; doi:10.1371/journal.ppat.1008666)
Supplement: S2 Table — (DOCX) [file ppat.1008666.s012.docx]

| **PCR fragment** | **Gene region** | **Primers** | **Sequence (5’ to 3’)** | **Fragment size (bp)** |
| --- | --- | --- | --- | --- |
| Fragment 1 | Rh13.1 | Forward | TCCTGCCGTACAAGAACGAC | 1,793 |
|  |  | Reverse | TCGGTCCGAATGTTTCCGTT |  |
| Fragment 2 | Rh61/Rh60 | Forward | TGGCTCGTCATCATCATCGG | 2,578 |
|  |  | Reverse | AGAGACGTCGTGTTTGGCAT |  |
| Fragment 3 | Rh152/Rh151 | Forward | GAAGTGCCGTACGGGTAGAG | 1,897 |
|  |  | Reverse | TGGGGTCCGCTCTTTTATGG |  |
| Fragment 4 | Rh157.5-Rh157.6 | Forward | TCTACCCGTCTCTCAGACCA | 2,981 |
|  |  | Reverse | GATCGAGTCTCAGGCCGATG |  |
| Fragment 5 | Rh158-Rh161 | Forward | AAGGCAGCTGAGAAGGAAGC | 2,993 |
|  |  | Reverse | CATCCATCAACATCACCGCG |  |
| Fragment 6 | Rh164 | Forward | TTGTCGTTGTAGGACCCAGC | 1,914 |
|  |  | Reverse | TTGGAAACGGCTGCGATACT |  |
| Fragment 7 | Rh167 | Forward | GTGTCGCGGATAGTAGCACA | 1,834 |
|  |  | Reverse | CGTTCTCTCAGCCATTCCGA |  |
| Fragment 8 | Rh197 | Forward | AACAGCATATGGGAGGGTGC | 1,956 |
|  |  | Reverse | ATTCCCCAGAGCATTTGCCA |  |

**Supplemental Table 3. Primers used to enrich eight gene-fragments in viral DNA samples isolated from the urine of FL-RhCMV/Rh13.1apt-inoculated RM.**
