# Supplementary material for: In vitro and in vivo characterization of a recombinant rhesus cytomegalovirus containing a complete genome
Source: PLoS Pathog. 2020 Nov 24;16(11):e1008666. doi: 10.1371/journal.ppat.1008666 (PMC7723282; doi:10.1371/journal.ppat.1008666)
Supplement: S3 Fig — A) Phylogenetic tree based on the protein sequences of NHP CMV genes homologous to HCMV UL83 encoding the major tegument protein pp65 from representatives of each NHP CMV species. B) ORF structure of the UL83 family genes in each NHP CMV species. Each gene is color-coded using the same colors as in A) showing the presence/absence of each ORF in a given NHP CMV species. (PDF) [file ppat.1008666.s003.pdf]

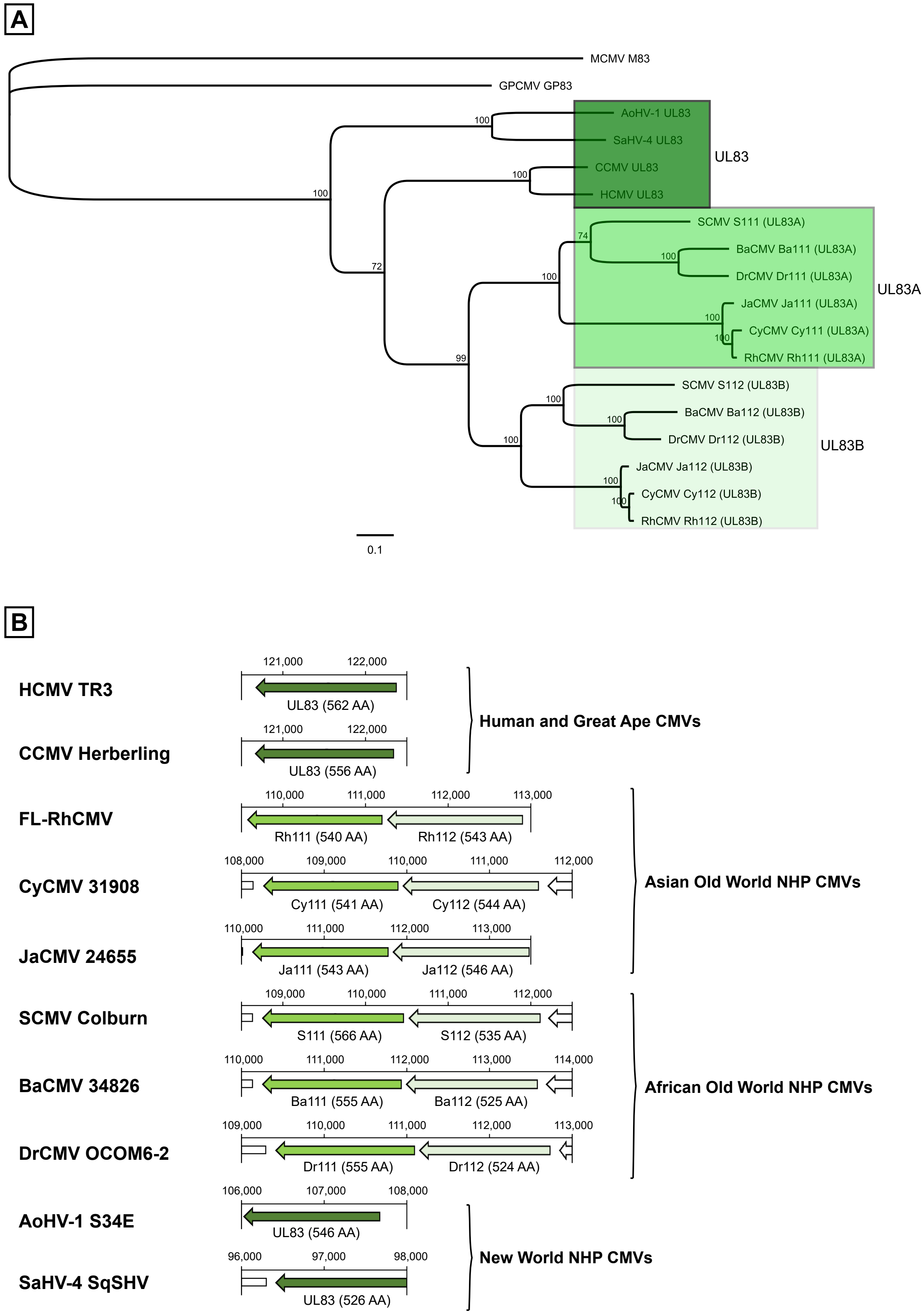

A
MCMV M83
GPCMV GP83
AoHV-1 UL83
SaHV-4 UL83
CCMV UL83
HCMV UL83
UL83
SCMV S111 (UL83A)
BaCMV Ba111 (UL83A)
DrCMV Dr111 (UL83A)
JaCMV Ja111 (UL83A)
CyCMV Cy111 (UL83A)
RhCMV Rh111 (UL83A)
UL83A
SCMV S112 (UL83B)
BaCMV Ba112 (UL83B)
DrCMV Dr112 (UL83B)
JaCMV Ja112 (UL83B)
CyCMV Cy112 (UL83B)
RhCMV Rh112 (UL83B)
UL83B
0.1
B
HCMV TR3
UL83 (562 AA)
CCMV Herberling
UL83 (556 AA)
Human and Great Ape CMVs
FL-RhCMV
Rh111 (540 AA)
Rh112 (543 AA)
CyCMV 31908
Cy111 (541 AA)
Cy112 (544 AA)
JaCMV 24655
Ja111 (543 AA)
Ja112 (546 AA)
Asian Old World NHP CMVs
SCMV Colburn
S111 (566 AA)
S112 (535 AA)
BaCMV 34826
Ba111 (555 AA)
Ba112 (525 AA)
DrCMV OCOM6-2
Dr111 (555 AA)
Dr112 (524 AA)
African Old World NHP CMVs
AoHV-1 S34E
UL83 (546 AA)
SaHV-4 SqSHV
UL83 (526 AA)
New World NHP CMVs
