# Supplementary material for: In vitro and in vivo characterization of a recombinant rhesus cytomegalovirus containing a complete genome
Source: PLoS Pathog. 2020 Nov 24;16(11):e1008666. doi: 10.1371/journal.ppat.1008666 (PMC7723282; doi:10.1371/journal.ppat.1008666)
Supplement: Attachment — Submitted filename: Rebuttal letter FL-RhCMV paper (final).pdf [file ppat.1008666.s014.pdf]

Response to the Reviewers

We would like to thank the reviewers for the time and effort they invested into evaluating our manuscript. We highly appreciate all the comments and constructive criticism and we have revised our manuscript according to their helpful suggestions as much as it was possible within the given timeline for resubmission.

Part I – Summary

Please use this section to discuss strengths/weaknesses of study, novelty/significance, general execution and scholarship.

Reviewer #1: In their manuscript titled "In vitro and in vivo characterization of a recombinant rhesus cytomegalovirus containing a complete genome," Taher and colleagues describe the repair of all the gene defects in RhCMV 68-1. Then, they characterize the full length RhCMV (FL-RhCMV) both in vitro and in vivo. In light of the potential value of CMV-based vaccines, it is important to understand how a fully intact CMV virus would replicate in rhesus macaques. Much of the vaccine work has been performed using the defective CMV viruses. While those pioneering studies greatly advance the field of infectious disease vaccine research, a more thorough understanding of how CMV can be manipulated to be both safe and immunogenic is necessary for vaccine development. By generating this full length rhesus CMV, engineering some deletions, and then assessing the impact of those, these authors have generated an essential reagent in the toolbox needed to develop a clinically relevant CMV-based vaccine.

We would like to thank the reviewer for her kind remarks.

These authors clearly demonstrate a large amount of work in this manuscript, and they should be commended for it. There are, however, several points in the text that need clarification. Right now, it is difficult to read, and I think that some of the key findings get buried behind other pieces of data that seem tangential.

Overall, this is a study that will provide a nice addition to the field. It is important to describe the characterization of a FL-RhCMV and identify the impact of specific mutations on function of this virus. This paper, however, feels like there is extra data included that is not relevant to a discussion of the construction of the virus. This paper would greatly benefit from removing extraneous data and telling a clear and linear narrative about how they made the FL-RhCMV, how it replicates, and then how specific mutations affect its function.

We believe that the reviewer is referring to the inclusion of phylogenetic analyses of RhCMV gene families in this manuscript. While we agree that this might appear tangential to the main story line in the manuscript we felt it was important to include these data to support the notion that we had indeed generated a RhCMV strain that was representative of a true wildtype strain. Furthermore, this analysis allowed us to refine gene annotations that will be helpful in the future for the design of specific mutations in the FL-RhCMV genome. We believe that the presented genomic data complements the storyline we are trying to present and we think that it will be of interest to a

substantial segment of the readership as it was for instance lauded by reviewer 2. Hence, we decided to keep it as supplemental information in the revised version of the manuscript. In addition, as described further below, we decided to include new data and new descriptions of the experimental details in the revised manuscript that will hopefully increase readability and comprehension.

Reviewer #2: In this paper by Malouli and colleagues, a recombinant BAC with repairs in a cluster of rhesus CMV genes is generated. The BAC construct restores virulence to the virus. This is a big advance in the study of RhCMV and will enable the study of pathogenesis in this important animal model. The paper will be of interest to basic scientists, molecular virologists, and translational scientists in internal medicine and pediatric infectious diseases.

The paper is well written and has appropriate controls. Conclusions are supported by the data. Inoculation of monkeys with the reconstituted virus resulted in significant replication in the blood similar to primary isolates of RhCMV and end-organ disease. Viral dissemination and viremia was greatly reduced upon deletion of genes also lacking in 68-1. Transcriptome analysis of infected tissues further revealed that chemokine genes deleted in 68-1 were highly expressed, consistent with an immune modulation effect.

It is presumed from this work that these genes PROMOTE inflammation which facilitates dissemination, and are not for IMMUNE EVASION per se. Thus, RhCMV, like human CMV, "loves an inflammatory party".

In addition to the usefulness of the new BAC and the insights it provides into pathogenesis, the paper is an elegant treatise on molecular phylogeny of CMVs and is very useful in-and-of-itself for this reason.

We thank the reviewer for the kind remarks.

A few suggestions are offered toward the goal of improving the paper:

1. It is a bit of a misnomer to speak of extensive "viremia in many tissues". Viremia is in the blood; virus in the tissue is in the tissue, not in the blood.

We agree with the reviewer's suggestion and changed this in the text from "viremia" to "viral genome copy numbers".

2. Lines 571-573, "...supernatants from cells that were spin-inoculated with the original urine sample were collected at full CPE and used to infect three T-175 flasks of primary rhesus fibroblasts". Recognizing the difficulty in amplifying CMV, even a single passage on fibroblasts could result in genome modifications and mutations. Did the authors try NGS directly from the urine sample? Can the authors provide reassurance that the passage did not modify the parental sequence?

The reviewer is correct that any culture of CMV can lead to selective growth of virus clones within a swarm or collection of clones found in a primary sample as well as immediate adaptation to

tissues culture. In fact, we did observed genome alteration in some isolates that are likely the result of early tissue culture adaption, like the loss of the Rh13.1 gene or its homologous genes in multiple isolates. Unfortunately, direct sequencing of entire virus genomes from tissue samples is not possible on any platform without prior target enrichment. While this has been established for HCMV we do not currently have this technology at our disposal. Nevertheless, comparing more than two dozen primary or cultured isolates allowed us to estimate the full coding potential of unaltered circulating isolates. Since it is unlikely that all isolates have acquired the exact same adaptation at the same time we were able to build consensus sequences across strains resulting in a complete ORFeome of a representative CMV isolate. Therefore, we are confident that we have generated a viral recombinant that exhibits most, if not all, characteristics of a primary, unpassaged isolate.

3. Lines 777-782, it seems surprising that given the exceptional sensitivity of real-time PCR that a nested step would be require. Did the authors fail to see real-time PCR signal with single-primer-pair real-time PCR? Did the authors attempt digital PCR? Some comment on why nested PCR was necessary would be useful.

CMV viral genome levels in tissues are generally low and the infection is not homogeneous, thus we must sample a large amount of the tissue in order to accurately quantify viral loads. Our nested PCR procedure allows us to analyze up to 50µg of total DNA for each sample; and this method is the most sensitive method that we have empirically determined for the detection of CMV in tissues. We have worked closely with ABI to develop a digital PCR assay, and while the method is highly accurate at low levels, we quickly realized that a major impediment with the digital PCR is that the sampling quantity of total DNA was inadequate and it was too costly to run the high number of chips necessary to test the same amount of DNA that we test using the nested PCR method.

4. Lines 225-227, "...Loss of RL13 could be prevented in HCMV by conditional expression of RL13 mRNA under the control of a tet operator (tetO) and growth in tet-repressor (tetR)-expressing fibroblasts." True, but the authors should also point out that changes in RL13 can be obviated by inclusion of antibody in the cell culture media (https://doi.org/10.3390/v11030221). The authors should cite this paper and included a comment on this.

We have included the suggested publication and added the following discussion of the findings to the results section of the manuscript:

“Loss of a functional RL13 protein was shown to result in more rapid cell to cell spread and increased cell free virus in the supernatant of infected cells suggesting that RL13-deficient HCMV mutants have a substantial growth advantage *in vitro* (44). This interpretation is supported by the finding that RL13 remains intact when cell free spread of a clinical HCMV isolate was prevented by adding CMV-hyperimmunoglobulin (HIG) to the culture medium (45).”

5. The differences in this novel, new BAC and the 68-1 construct seem clear and well delineated in this paper. It is unclear what the authors believe the role of these UL128-131 homologs in the RhCMV genome really is. The investigative group has published that these ORFs are involved in the generation of novel, new "promiscuous" T cell epitopes; that they form a pentamer that is important in epithelial cell entry; and that there is a chemokine function attributable to some

regions of this cluster of gene. It would be helpful if the authors could provide a summative, integrative statement in the discussion as to what function they believe is most important, and the pros/cons of considering this as a truly homologous cluster to HCMV.

The role of the UL128-131 subunits in RhCMV pathogenesis and immunology is indeed complex. In the context of this manuscript the repair of UL128-UL131 results in increased cell tropism and thus likely contributes to increased dissemination and replication *in vivo*. As further evidence for a functional reconstitution of the PRC in our FL RhCMV we now include new data showing that entry of PCR-intact FL-RhCMV and 68-1.2 RhCMV into rhesus epithelial cells can be blocked by an N-terminal peptide of the previously described PRC receptor on host cells: the G protein coupled olfactory receptor OR14I1. In addition, we previously reported that deletion of UL128 and UL130 is important for the induction of unconventionally MHC-restricted $CD8^+$ T cells. The exact role of each of these subunits in controlling T cell responses goes beyond the scope of the current manuscript and is the topic of a forthcoming manuscript. However, we now include a brief discussion of the complex role of these subunits in tropism and immune programming in the discussion section:

"In the guinea pig model it was shown that the repair of the PRC lead to a more pathogenic virus capable of congenital transmission to pubs in pregnant dams (70), likely as the result of increased systemic viral loads during the course of infection. It is furthermore possible that a PRC-independent function of the UL128 and UL130 homologs Rh157.5 and Rh157.4 could play an important role during the early stages of infection. The N-termini of both proteins show homology to host CC and CXC chemokines respectively and chemokine function has been shown for HCMV UL128 in *in vitro* chemotaxis assays (71). Additionally, we observed that vaccine vectors based on 68-1 elicited $CD8^+$ T cell responses that were unconventionally restricted by MHC-II and MHC-E classical MHC-Ia alleles whereas repair of Rh157.5 and Rh157.4 in 68-1.2 RhCMV resulted in the induction of conventionally MHC-I-restricted $CD8^+$ T cells (14, 18, 72). Conceivably, the unconventionally restricted $CD8^+$ T-cell responses induced by 68-1-like RhCMV might be more efficient in controlling virus replication *in vivo* compared to conventionally restricted $CD8^+$ T cells elicited by wildtype RhCMV."

6. The authors comment on animal models (line 485) for congenital CMV infection. It is agreed that this is a very important off-shoot of this work. Toward providing a more comprehensive and current list of references, the authors should add this reference to their paper and reference it here: DOI: 10.1093/infdis/jiz484

We have included the proposed review into our manuscript at the suggested location.

7. Also along these same lines, it is surprising that the paper does not comment on this paper: doi: 10.1172/jci.insight.94002. In this paper, Nelson et al. The paper is cited but not commented on. In this paper, macaques that were inoculated with fibroblast-tropic 180.92 and epithelial cell–tropic UCD52/UCD59 had disease. Can Malouli and colleagues comment on the pathogenicity of UCD52/UCD59 compared to their fully wild-type BAC virus? If UCD52/UCD59 can cause disease, including congenital infection, what advantages does this full length BAC virus offer?

We thank the reviewer for pointing this out and we have added the paragraph shown below to our discussion regarding the implications of our cloned full-length virus for this model. In fact, we are collaborating with the Permar team to examine fetal transmission of the FL-RhCMV and preliminary data support the conclusion that FL-RhCMV is as pathogenic as the previously published UCD52/59/180.92 mix. However, the results are too preliminary to include in the current manuscript. In order to define viral determinants of pathogenesis and fetal transmission it is necessary to introduce defined genetic modifications into the RhCMV genome. Up to now this has not been possible since UCD52/59 and 180.92 are not cloned. The main reason for generating FL-RhCMV was to enable such experiments. We have now added an additional paragraph to the manuscript to emphasize the importance of FL-RhCMV for the congenital infection model.

"The *in vitro* and *in vivo* characteristics of FL-RhCMV described here are consistent with this virus being representative of wildtype RhCMV. Based on these observations we anticipate that this recombinant will be useful for RM models of CMV pathogenesis, such as the fetal inoculation model as well as a model of congenital infection (74-76). Up to now these models relied on low passage RhCMV isolates such as UCD52 and UCD59 which are non-clonal and are difficult to modify genetically. The availability of a true wildtype clone that is amenable to BAC recombineering will help to define viral determinants of congenital infection as well as help unravel viral immune evasion strategies that limit the efficacy of vaccines or hyperimmune globulin (HIG) treatment (27) to prevent congenital infection or pathogenesis in immunocompromised hosts."

Reviewer #3: Taher, Mahyari, Malouli and colleagues report about a novel BAC-cloned RhCMV which they have constructed on the basis of the well-established 68-1 strain by restoring a number of deviations that this strain has as compared with primary RhCMV isolates. In detail, the authors repaired Rh13.1(RL13 in HCMV) Rh61/Rh60 (UL36), Rh152/Rh151 (UL119/UL118), Rh157.5 and Rh157.4 (UL128 and UL130), Rh164 (UL141), Rh167, Rh197 (US14D), and this restored RhCMV-BAC they named "full length Rhesus cytomegalovirus" FL-RhCMV. With this work they not only complemented the BAC cloned 68-1 strain on a genetic level, but apparently they also restored biological functions in a way that the new clone resembles primary isolates with regard to in vivo replication in rhesus macaques. This is an outstanding contribution to the field, given the virus is made available to the scientific community without restrictions.

We thank the reviewer for his/her kind remarks. We're happy to share the FL-RhCMV BAC with the scientific community after publication of this manuscript.

Part II – Major Issues: Key Experiments Required for Acceptance

Please use this section to detail the key new experiments or modifications of existing experiments that should be absolutely required to validate study conclusions.

Generally, there should be no more than 3 such required experiments or major modifications for a "Major Revision" recommendation. If more than 3 experiments are necessary to validate the study conclusions, then you are encouraged to recommend "Reject".

Reviewer #1: 1. Figure 1 shows all the different repairs that were made in 68-1 to generate the FL-RhCMV. The description of the repair steps are in the legend, but they are not explained in the text. The rationale for making each repair is buried. If each repair is important, then a rationale explaining each repair mutant and why only certain mutants were tested in culture would be useful.

We thank the reviewer for pointing this out. We now describe each repair step and the possible function of the repaired genes in the corresponding text of the results section. As described in the text, our strategy was to generate a wildtype-like virus by reconstituting a complete ORFeome using partially repaired clone 68-1.2 as our starting point. However, the importance of each repaired ORF for infection and pathogenesis can only be determined by "un-repairing" individual ORFs and examining the resulting viral mutants *in vivo*. This was partially accomplished by deleting the UL128-130 and UL146-147 homologous genes and monitoring the *in vivo* spread as shown. However, additional future experiments that go beyond the scope of the current manuscript will be required to evaluate the role of other repaired genes (e.g. viral Fc receptors or apoptosis inhibitors) for RhCMV infection and pathogenesis.

2. Several mutations are made in the FL-RhCMV backbone to recapitulate some of the specific deletions in 68-1. While this approach makes a lot of sense, this is not laid out very clearly. A summary of all the different mutations in FL-RhCMV and their rationale would be beneficial. This includes the deletion of Rh13.1 and the various sub mutations or insertions into that backbone. Each of these mutants seems to be just a little bit different and their purposes are different. It seems like a variety of mutations were made (for good reasons) and then just added together in this paper. A linear discussion of why these mutations were made and what hypothesis each one tested is needed for the reader to better understand the manuscript.

We apologize for the number of similar sounding mutants and agree that this can be confusing. To improve readability, we are now highlighting the differences between the individual vectors used by including schematics into the figure where they first appear. Overall, we have created seven different constructs in this manuscript, not counting the published BAC derived RhCMV constructs 68-1 and 68-1.2. Six of these vectors simply present different solutions to overcome the experimentally confirmed *in vitro* genome instability in FL-RhCMV due to a now fully functional Rh13.1 (RL13) ORF. The different constructs shown either represent a full deletion of the ORF, techniques to transiently degrade the encoded mRNA to avoid selection against the ORF or replacing the ORF with an immunological markers. Additionally, we also constructed a deletion of Rh157.5 (UL128) and Rh157.4 (UL130) furthermore containing a deletion of Rh158-Rh161 (UL146 family) based on a FL vector carrying a Tb6Ag transgene in the Rh13.1 locus to stabilize the viral genome and to serve as an immunological marker. This mutant was generated as a first step to investigate the role of each repaired ORF in viral dissemination and pathogenesis as discussed above. The underlying rationale for this mutant has now been expanded in the text of the results section.

3. The transcript analysis seems like a herculean amount of work, but I cannot tell how that impacts our understanding of the construction of the full length RhCMV. This needs to be explained more clearly. Was this section intended to measure expression of CMV genes or assess how the CMV genes would modulate host immunity?

This experiment was enabled by our finding that FL-RhCMV replicated to high levels in some tissues. As such it represented an additional quality control of our FL-RhCMV construct. In particular, this experiment allowed us to compare expression of the repaired gene regions *in vitro* and *in vivo*. Quite surprisingly, we observed that some of the repaired chemokine-like genes were among the most strongly expressed genes both *in vitro* and *in vivo*. While the reason for this high level of expression requires further exploration, it strongly indicates a prominent role of the corresponding gene products in immune modulation. Thus, while we did not set out to use this experiment in the context of immune evasion, the results provided a possible lead that will be investigated further in the future. To further clarify the rationale we changed the introduction to the relevant paragraph as follows:

"The high genome copy numbers measured in several tissues of FL-RhCMVΔRh13.1/TB6Ag-inoculated RM at 14dpi provided an opportunity to compare viral gene expression from a fully characterized viral genome by RNAseq analysis *in vivo* and *in vitro*. In particular, this analysis would allow us to simultaneously verify expression of all genes encoded in the repaired loci."

4. The phylogenetic relationship between FL-RhCMV and other NHP CMV strains also seems inessential for this study.

Please see our response above.

Reviewer #2: This reviewer has no criticism of any experiments nor any new suggestions for new experiments. This is elegant and well-controlled work.

We would like to thank the reviewer for his/her positive evaluation of our work.

Reviewer #3: The data presented in Fig. 5A are very surprising, given the strong inhibitory effect of Rh13.1 shown in figure 4B that also suggested an effect on cell-free virus spread (note the lack of dispersed infected cells).

Hence we doubt whether FL-RhCMV in the experiments of 5A had an intact ORF Rh13.1 during the complete course of the growth curve. In HCMV, it is known that RL13 already is disrupted during reconstitution of the virus by transfection of the BAC. It is conceivable that Rh13.1 became disrupted during initial replication and was selected during the second and/or third replication cycle of this multistep growth curve.

We agree with the reviewer's interpretation of the results and we have altered the corresponding text in the results section as follows:

"It was previously shown that repair of the PRC increased the ability of 68-1.2 RhCMV to infect epithelial and endothelial cells without affecting growth characteristics in fibroblasts (25). Consistent with these earlier reports we observed that growth characteristics of PRC-repaired, but Rh13.1-deficient, 68-1.2 were comparable to that of 68-1 in rhesus fibroblasts with respect to kinetics and peak titers in a multistep growth curve (**Fig. 5A**). Interestingly, FL-RhCMV/Rh13.1/apt also grew similar to 68-1 in fibroblasts despite Rh13.1 expression in the

absence of tetracycline. Given the impact of Rh13.1 expression on viral spread observed above, this likely indicates rapid mutation of the Rh13.1 gene during multistep growth conditions."

The authors must at minimum sequence the virus harvested on day 12 to check whether Rh13.1 was still intact.

Unfortunately, these samples are no longer available and it would thus require us to repeat the entire experiment. However, we feel this is unnecessary since other data show in the manuscript already demonstrate that Rh13.1 is functional (e.g. Fig. 4A, B) and that it rapidly mutates (Fig. 4C).

This is particularly important in the context of the authors' remark that "our results indicate that FL-RhCMV is remarkably similar to low passage clinical isolates of HCMV with respect to growth in tissue culture, tissue tropism and genetic stability in vitro." (lines 285-287). HCMV isolates grow strictly cell-associated in relatively small foci, which resembles FL-RhCMV in TRF (Fig 4B) but is in sharp contrast to the strong cell-free growth in Fig 5A.

We have added a statement to support this interpretation of our results as shown above.

Similarly, sequence data should be obtained regarding the samples in in-vivo experiments to check whether the restored gene regions are still intact. In my opinion, this is at least mandatory for those gene regions which are functionally addressed in this paper, i.e. Rh13.1; the PRC genes, and so forth.

We are grateful to the reviewer for suggesting these experiments. We performed qPCRs with primer/probe sets designed to target Rh13.1 (RL13) and Rh157.5 (UL128) on DNA derived from plasma, saliva and urine samples from infected animals. The comparison to qPCR results obtained using an established IE gene primer/probe set can then be used to determine whether Rh13.1 or Rh157.5 is under-represented in any of the samples which would indicate gene deletions. As shown in the updated Fig.6 this experiment revealed that Rh13.1 and Rh157.5 ORFs were not deleted at any time during the initial 65 days of the experiment.

Since this approach cannot detect more subtle changes we further investigated potential mutations in all repaired regions by deep sequencing PCR fragments obtained using primer sets flanking the repaired regions. As shown in new supplementary Fig. 8, using this approach we did not detect any sequence variations compared to the consensus sequence of the original BAC clone for six of our eight DNA fragments. Both exceptions were highly interesting. For Rh13.1 we observed that a small percentage of the genomes of the inoculum displayed a deletion. However, this was not detected *in vivo* in all three animals, strongly indicating that *in vivo* replication selects for intact Rh13.1 in contrast to *in vitro* replication that selects against it.

In contrast, we observed deletions in the PCR fragment ranging from Rh59 (UL35) through Rh61/Rh60 (UL36) to Rh66/Rh62 (UL37), both in the inoculum as well as in genomes shed by one of the animals. This is somewhat unexpected as this genomic region encodes inhibitors of apoptosis, namely homologous of UL36 (vICA) and UL37x1 (vMIA), in HCMV. Since *in vivo* data generated in the mouse model using MCMV indicates that deletion of the homologous ORFs leads to *in vivo* attenuation of the virus, we would assume the same to be true for RhCMV. Conceivably, our results could indicate that the RhCMV ORFs do not share the exact same

function and *in vivo* importance of their HCMV or MCMV counterparts. It is also possible that the deletion mutants acquired during the brief initial tissue culture during virus stock preparation are either complemented by a wildtype virus through co-infection during their initial replication or that they will be eventually lost from the *in vivo* population. Longer term studies might be required to resolve this issue

In addition, we detected a sizable RhCMV population in one of the urine samples carrying a synonymous point mutation in Rh66/Rh62 (UL37). This silent mutation is not present in the inoculum and thus possibly reflects a benign genomic alteration without substantial viral fitness cost. Taken together, these new data strongly support the conclusion that BAC derived FL-RhCMV is stable *in vivo*, particularly in Rh13.1 and the genes encoding for pentamer subunits. Thus, while these gene products are dispensable, or even detrimental, for growth *in vitro* they likely play an important role during infection.

Part III – Minor Issues: Editorial and Data Presentation Modifications

Please use this section for editorial suggestions as well as relatively minor modifications of existing data that would enhance clarity.

Reviewer #1: There are typos and acronyms not spelled out.

We thank the reviewer for pointing this out and we corrected these mistakes throughout the manuscript.

Reviewer #2: See comments above. Generally, exceptionally well written and clear manuscript.

We are grateful to the reviewer for his very favorable evaluation of our manuscript.

Reviewer #3: 216: "is likely a representative of the genomes contained in the original 68-1 isolate" should be further toned down, as the original sequence is not available for comparison => e.g. "expected to resemble" "expected to approximate"

We altered the manuscript according to the reviewer's suggestion.

249: "mutations in the Rh13.1 homologs found in many old world NHP CMV genomes (Fig. 3) are due to"; this reference to Fig. 3 does not make much sense as no sequence data are shown there.

Although it is correct that we do not show sequencing data in Fig.3, we highlighted ORFs that have acquired changes resulting in elongated or truncated ORFs in a different color. This indicates that Rh13.1 is more often mutated than other ORFs consistent with Rh13.1 mutations resulting from tissue culture adaptation. While we believe our statement to be true, we can understand how it can be confusing to the reader, hence, we have decided to remove the reference.

259: Typo: "we cannot observed a phenotype" => observe

We thank the reviewer for pointing out this mistake which we have corrected in the revised manuscript.

264: "In contrast, a FL-RhCMV vector carrying an SIVgag insert replacing Rh13.1 (FL-RhCMVΔRh13.1gag) displayed an increased ability to enter RPE cells compared to 68-1." We miss data and/or information regarding this virus.

We apologize for not providing more detailed information describing the mutant we have used in this experiment. We have updated the figure by including a schematic showing the altered genomic region in this construct.

In Figure 5A, I do not find it accurate to show these sem bars. Obviously, they are the result of two technical replicates from two biological replicates, which must not mixed to yield "n=4" in my opinion. So, I suggest to leave it with means of two biological replicates. If the authors really want to improve these data, they may add one further biological replicate. As the data appear at the moment, there might be a significant difference (about 10fold) between 68-1 and 68-1.2, which can, however, only be tested after an additional experiment. NOTE: this remark only applies if sequence data show that RH13.1 and the PRC gene region are intact in the repaired versions, see "major issues".

We agree and we have changed the figure following the reviewer's suggestions. We now show the arithmetic mean of the two independent biological repeats without error bars.

In Figure 6, panel C does not add information but is rather confusing, due to the fact that the last two time points of "A" are no triplicates. Hence the "indentation" of the curve at around 18 days is meaningless. I suggest to remove this panel and rather include all six curves in one graph, with the curve sets of "A" and "B" in different colors.

As mentioned above we have completely reworked the entire figure to include additional data. The revised figure displays viral genome copy numbers detected in plasma, saliva and urine samples from all examined animals for up to 70 days post infection. Animals that received different inoculums are shown in different colors as suggested by the reviewer. Finally, we removed the mean value altogether since it did not add additional information.
